# Transactive memory systems scale for couples: development and validation

**DOI:** 10.3389/fpsyg.2015.00516

**Published:** 2015-05-05

**Authors:** Lauren Y. Hewitt, Lynne D. Roberts

**Affiliations:** School of Psychology and Speech Pathology, Curtin University, Perth, WA, Australia

**Keywords:** transactive memory, romantic couples, memory research

## Abstract

People in romantic relationships can develop shared memory systems by pooling their cognitive resources, allowing each person access to more information but with less cognitive effort. Research examining such memory systems in romantic couples largely focuses on remembering word lists or performing lab-based tasks, but these types of activities do not capture the processes underlying couples’ transactive memory systems, and may not be representative of the ways in which romantic couples use their shared memory systems in everyday life. We adapted an existing measure of transactive memory systems for use with romantic couples (TMSS-C), and conducted an initial validation study. In total, 397 participants who each identified as being a member of a romantic relationship of at least 3 months duration completed the study. The data provided a good fit to the anticipated three-factor structure of the components of couples’ transactive memory systems (specialization, credibility and coordination), and there was reasonable evidence of both convergent and divergent validity, as well as strong evidence of test–retest reliability across a 2-week period. The TMSS-C provides a valuable tool that can quickly and easily capture the underlying components of romantic couples’ transactive memory systems. It has potential to help us better understand this intriguing feature of romantic relationships, and how shared memory systems might be associated with other important features of romantic relationships.

## Introduction

Romantic relationships confer many benefits to their members; for instance, people in romantic relationships experience boosts to self-esteem, life satisfaction and happiness (Dush and Amato, 2005), and report experiencing benefits such as social and emotional support, companionship, sexual gratification, intimacy, and security (Sedikides et al., 1994). One benefit that may be less apparent is the opportunity for romantic partners to pool their cognitive resources, enabling them to have access to more information, but with less cognitive effort required from each member of the pair. In other words, people in romantic relationships might develop shared transactive memory systems (Wegner et al., 1985; see also Harris et al., 2014).

### Transactive Memory Systems Theory

Wegner and colleagues used the term “transactive memory systems” to describe the way that people in relationships use each other’s memories as extensions of their own (Wegner et al., 1985; Wegner, 1987). Transactive memory systems develop when people share responsibilities and learn about each other’s expertise; people might share transactive memory systems within intimate relationships, family groups, friendships and even work groups (Wegner et al., 1985; Wegner, 1987; Hollingshead, 1998a,b, 2000). In close relationships, because people know so much about each other, they are able to access the information the other people know and as a result can boost their collective memory performance while saving on individual cognitive effort. For example, one person in the relationship might be good at remembering directions but terrible at remembering phone numbers. On the other hand, the other person may be good at remembering phone numbers, and terrible at remembering directions. If each person knows the other’s areas of expertise (and non-expertise), they can use each other in the same way that people use diaries, alarms and other memory aids to remember things that they would not remember on their own (Wegner et al., 1985; Wegner, 1987; Stephenson et al., 1991). Additionally, because people know about each other’s expertise (and non-expertise) they can make judgments about the reliability and value of any information conveyed. It is easy to see how a shared transactive memory system might work to help couples navigate their daily lives with ease and efficiency. In fact, Wegner et al. (1991) go so far as to suggest that the transactive memory system is so beneficial to couple’s daily lives, that if the relationship ends, the loss of the shared transactive memory system may be at least partly responsible for a period of poor functioning and confusion following the relationship breakdown. Similarly, Harris et al. (2014) suggest that a close lag in cognitive decline found in elderly couples could be due to the deterioration of the couples’ shared memory system; if one member of the memory system experiences a cognitive decline, it consequently affects the couple’s shared memory system, and thus the other member’s memory suffers too.

There is evidence to support the idea that people in intimate relationships can benefit from transactive memory systems (Wegner et al., 1991; Hollingshead, 1998a,b). For example, Wegner et al. (1991) recruited couples who had been together for at least 3 months, and found that they used each other as extensions of their own memories, outperforming pairs of strangers at remembering category exemplars in different areas of expertise (for example science, food, spelling). Similarly, Hollingshead (1998a,b) demonstrated that couples who worked together outperformed pairs of strangers who worked together at both general knowledge tests and remembering word lists. One limitation of research in this area is that it typically focuses on memory performance using lab-based tasks; researchers infer that transactive memory systems exist on the basis of comparisons between participants’ coordinated recall (with his or her romantic partner) and participants’ individual recall, or recall with a stranger (Lewis, 2003). Although these comparisons demonstrate that coordination can improve memory performance, the research focuses on simple declarative memory tasks, and often require one specific correct solution (Lewis, 2003); these tasks may not capture the complex or subtle ways in which couples use their transactive memory systems, nor the processes underlying such a memory system. In addition, the research examines memory for only a small range of topics (for example, history, math, spelling), which may not be representative of the range of topics, tasks, and decisions that couples’ use their transactive memory systems for in everyday life (Wegner et al., 1991). In addition, these lab-based tasks often require one specific correct solution (Lewis, 2003), and do not shed any light on the underlying processes involved when a romantic couple uses their shared memory system to negotiate complex tasks and decisions in their everyday lives.

In sum, it is not clear whether the transactive memory systems inferred from the previous research can be generalized beyond the specific experimental declarative memory tasks to the everyday situations that couples typically negotiate and work through together, nor what processes might underpin such a shared memory system. In addition, in a practical sense, relying on such time intensive measures of transactive memory makes it difficult for researchers to examine whether and how transactive memory systems might play a role in effects found in other research paradigms, especially if participation already requires considerable time and effort. In order to better understand the role, effects and processes underlying transactive memory systems in romantic couples, we addressed these challenges by developing and conducting an initial validation of a self-report measure of transactive memory in romantic couples.

### Lewis’s (2003) Self-report Measure of Transactive Memory

Lewis (2003) developed a task-independent self-report measure of transactive memory systems for use in organizational work groups. The transactive memory systems scale (TMSS; Lewis, 2003) assesses the three components of transactive memory systems: *specialization*—the extent to which members of the group have unique knowledge, *credibility*—the extent to which members of the group see the other members’ knowledge as credible, and *co-ordination*—the extent to which members are able to work together and access each other’s expertise. The measure is internally consistent, and has established convergent, discriminant and criterion validity (Lewis, 2003). We adapted Lewis’s (2003) TMSS to be appropriate for a sample of romantic couples (TMSS-C), using an initial draft adaptation by Stewart (2011). We conducted an initial validation of the TMSS-C by examining the factor structure of the instrument and by drawing comparisons with several theoretically related and unrelated measures to test it’s convergent and discriminant validity.

### Convergent Validity

Members of shared transactive memory systems have specific areas of expertise to concentrate their cognitive effort on, and they know what others’ areas of expertise are so they can access information from those areas. Members must not only know who knows what, but also be able to effectively coordinate their knowledge, so that everyone can access the information available. In order to examine the convergent validity of the TMSS-C, we compared participants’ responses on the TMSS-C with a measure of expertise coordination (Faraj and Sproull, 2000). If responses on this measure were associated with responses on the TMSS-C, it would provide evidence of the convergent validity of the TMSS-C as a measure of transactive memory systems in romantic couples.

### Divergent Validity

We examined divergent validity against two constructs: (1) reliance on memory aids, and (2) communication competence. Research has shown that people who have little faith in their own memory abilities have a greater tendency to rely on memory aids (e.g., diaries; Herrmann, 1984). Perhaps an alternative explanation for what appears to be a transactive memory system is that people who report relying on other people’s knowledge and memories have little faith in their own memory abilities, and as a result, tend to rely on memory aids, including—but not limited to—other people whom they have important relationships with. To examine whether the TMSS-C measures something beyond the tendency to rely on other people as external memory aids, we compared participants’ responses on the TMSS-C with a measure of participants’ reliance on memory aids (The *Mnemonics Usage* factor of the Memory functioning questionnaire; MFQ; Gilewski et al., 1990).

Communication is another key element of transactive memory systems; people who are more comfortable communicating may be better able to coordinate their output with other members of a transactive memory system, and may therefore be more likely to demonstrate the use of a transactive memory system. However, communication alone should not be able to fully explain the existence of a transactive memory system, because such a system also requires that the members have specialized areas of expertise, and knowledge of who knows what. To examine whether the TMSS-C measures something beyond communication skills, we compared participants’ responses on the TMSS-C with a measure of participants’ communication competence. If these two measures are not strongly associated with responses on the TMSS-C, it would provide evidence of the divergent validity of the TMSS-C as a measure of transactive memory systems in romantic couples.

We developed four specific hypotheses for this research:

H1: a higher order three-factor model mirroring the factor structure of the original TMSS would provide better fit to the data than a one-factor model, three-factor uncorrelated model or three-factor correlated model;

H2: there would be a moderate to large positive correlation between expertise coordination and the coordination subscale on the TMSS-C;

H3: there would be a weak to moderate correlation between the Mnemonics Usage factor of the MFQ scores and TMSS-C total scores;

H4: there would be a weak to moderate correlation between Self-Perceived Communication Competence Scale scores and TMSS-C total scores.

The research was conducted in two phases. In the first phase data was collected for the initial validation of the TMSS-C, and in the second phase data was collected to establish the test–retest reliability of the measure.

## Phase 1—Validation

### Materials and Methods

#### Participant Characteristics

In total, 397 people (297 (74.8%) women, 99 (24.9%) men, 1 unstated), aged 17–60 years (*M* = 26 years, SD = 9) took part. To be eligible to participate, people were required to identify themselves as being in a romantic relationship (of any sexual orientation) of at least 3 months duration. Participants reported being involved in different types of relationships including dating couples (42.2%), engaged couples (7.1%), defacto couples (22.7%), and married couples (27.5%). Forty-five respondents reported that their partner had already completed the survey.

#### Sampling Procedures

Participants were recruited through convenience and snowball sampling, using the researchers’ online and offline networks, posting on survey websites and online noticeboards, advertising on university and public noticeboards. Participants who completed the survey were given the opportunity to enter a prize draw to win a $100 Amazon.com voucher. In addition, some participants were recruited through Curtin University’s School of Psychology and Speech Pathology research pool and received course credit for their participation.

#### Research Design

A cross-sectional correlational design was used to assess the factor structure, internal reliability and validity of the TMSS-C.

#### Measures

An online questionnaire was constructed containing the TMSS (Lewis, 2003) adapted for couples (TMSS-C), the Knowledge Organization Questionnaire (Wegner et al., 1991), Faraj and Sproull’s (2000) measure of expertise coordination, the Self-Perceived Communication Competence Scale (McCroskey and McCroskey, 1988), the Mnemonics Usage factor of the MFQ (Gilewski et al., 1990), the Reactions to Research Participation Scale (Newman et al., 2001), and single item measures of demographics (age, gender, gender of partner, type of relationship, length of relationship, and shared activities).

***Transactive memory systems scale for couples***

**FIGURE 1:**
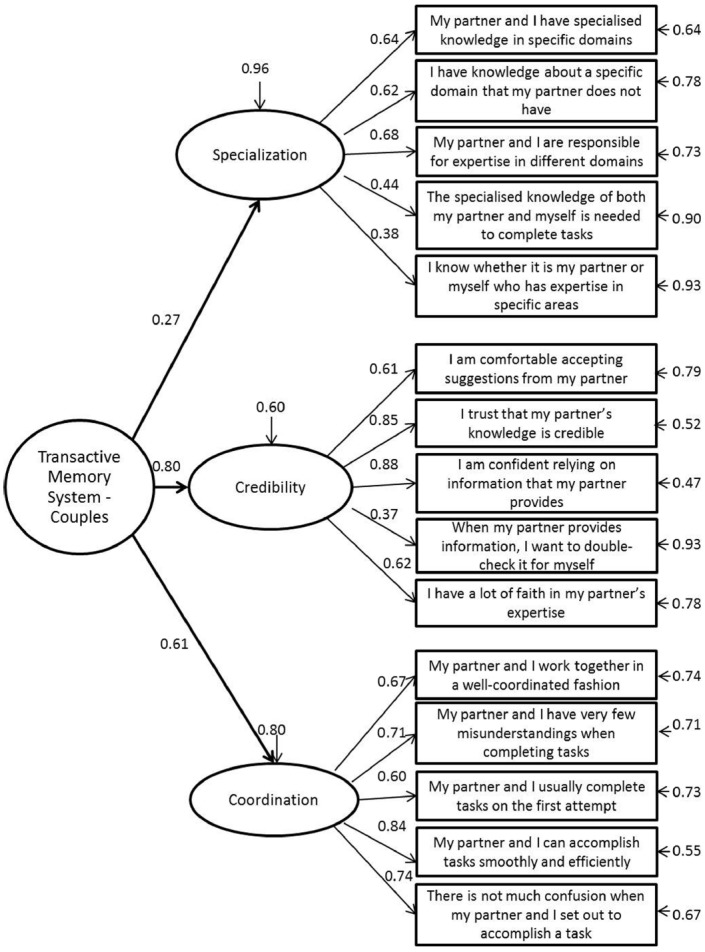
**Confirmatory factor analysis of TMSS-C**.

This measure was adapted from the 15-item TMSS by Lewis (2003) assessing the three components of transactive memory systems: specialization, credibility, and co-ordination (see Figure 1 for wording of each of the 15 final TMSS-C items). Participants responded to each statement using a five-point scale (1 = strongly disagree, 5 = strongly agree). Stewart (2011) adapted the wording of the original scale for use in research with romantic couples. The most common rewording was changing references such as “team member” and “our team” to more appropriate references such “my partner” or “my partner and myself.” Stewart also followed Lewis’s (2003) suggestion to reword the four reverse-coded items. For example, the original item “I did not have much faith in other members” “expertise.” was reworded to “I have a lot of faith in my partner’s expertise.” For the purposes of this study, Stewart’s adaptation has been further developed. One reverse-coded item, “When my partner provides information, I want to double-check it for myself,” was retained for clarity of wording.

***Expertise coordination***

Faraj and Sproull (2000) designed this scale to gage expertise coordination in work groups, with the idea that in order to benefit from expertise, work teams must be able to coordinate sharing information. The scale comprises 11 items that relate to each of three factors (Expertise Location, Expertise Needed, and Bring Expertise To Bear). We reworded the items to be appropriate to romantic couples. For example, the original item “The team has a good *map* of each other’s talents and skills” was adapted to “My partner and I have a good idea of each other’s talents and skills.” The three items that make up the Expertise Needed factor seemed inappropriate for romantic couples so this factor was excluded from our survey (e.g., “Some team members do not have the necessary knowledge and skill to perform well—regardless of how hard they try”). Faraj and Sproull (2000) demonstrated internal consistency with Cronbach’s alphas above 0.81 for all factors, and found that the measure was associated with work teams’ performance. In this sample the Expertise Location scale demonstrated good internal reliability (α = 0.87) and the Bring Expertise to Bear scale acceptable reliability (α = 0.60).

***Self-perceived communication competence scale (SPCC scale; McCroskey and McCroskey, 1988)***

This measure comprised 12 items; participants rated their competence at communicating in a variety of situations (0 = completely incompetent and 100 = completely competent). For example, one item read “*present a talk to a group of strangers.*” Consistent with previous research, the scale was found to have a high internal reliability (Cronbach’s alpha = 0.92; McCroskey and McCroskey, 1988; Richmond et al., 1989; Chesebro et al., 1992; Rosenfeld et al., 1995).

***Memory functioning questionnaire (Gilewski et al., 1990)***

The full MFQ is made up of 64 items that fit into four factors; research supports the four-factor structure of the MFQ on different aged samples and over time (Gilewski et al., 1990). Scores on the MFQ have been found to be associated with other self-report memory questionnaires (e.g., Hertzog et al., 1989), self-reported memory failures, performance on memory tests, as well as spouse’s ratings of each other’s memory functioning (Zelinski et al., 1990), providing evidence of the validity of this measure. Only the *Mnemonics usage* factor was included in the current research; this factor is measured by eight items in which participants rate how often they use a variety of reminders (for example, “*keep an appointment book*”). Ratings are made on seven-point Likert scales ranging from 1 (*always)* to 7 (*never)*. This factor showed a high internal reliability in the current sample (Cronbach’s alpha = 0.79).

#### Procedure

Prior to the research commencing approval was obtained from Curtin University Human Research Ethics Committee. The online questionnaire was hosted on Curtin University’s Qualtrics site (http://curtin.qualtrics.com). It was “sandwiched” between a participant information sheet and a debriefing page, both hosted by Curtin’s School of Psychology and Speech Pathology website, in line with best practice recommendations (Allen and Roberts, 2010). After completing the survey, participants were asked if they would like to participate in the second stage of the study; if they agreed an email contact address was requested for follow up.

The data from the Phase 1 survey was downloaded from Qualtrics into SPSS for analysis. In total, 397 cases were retained for analysis. There were an additional 152 cases that were ineligible; these cases were deleted because respondents either reported a relationship of less than 3 months, did not complete all items in the TMSS-C measure, or did not complete at least half the items in each of the scale measures, leaving a final sample of 397 for analysis. Missing data for scale items (MFQ 8 data points; Expertise Coordination 3 data points; and SPCC 17 data points) was replaced using the Expectation Maximization algorithm, an appropriate technique for small amounts of missing data (Tabachnick and Fidell, 2013).

### Results and Discussion

The descriptive statistics for all measures are presented in Table 1. As the table illustrates, mean ratings on the TMSS-C tended toward the upper end of the scale, suggesting that people in the sample made use of transactive memory systems in their romantic relationships. Mean ratings of both expertise location and expertise usage on Faraj and Sproull’s (2000) measure of expertise coordination were both reasonably high. Finally, on average, participants rated themselves as being reasonably competent at communicating in different situations, and as not particularly reliant on memory aids.

**TABLE 1:** **Descriptive statistics for scale measures (*N* = 397)**.

**Scale**	**Mean(SD)**	**Possible range**	**Actual range**
TMSS specialization	3.99(0.54)	1.00–5.00	2.00–5.00
TMSS credibility	3.99(0.57)	1.00–5.00	1.80–5.00
TMSS coordination	3.73(0.66)	1.00–5.00	1.20–5.00
Willingness to assign expertise	16.35(4.66)	0–26.00	0–26.00
Expertise assignment to partner	6.91(3.10)	0–26.00	0–16.00
Expertise location	4.24(0.62)	1.00–5.00	1.25–5.00
Bring expertise to bear	4.22(0.66)	1.00–5.00	2.00–5.00
Self-perceived communication competence	73.24(16.71)	0–100.00	12.50–100.00
Mnemonic usage	3.19(1.15)	1.00–7.00	1.00–6.63

#### Factor Structure of the TMSS-C

As an initial validation of the TMSS-C measure, we first examined whether it had the same factor structure as Lewis’s (2003) TMSS. Confirmatory factor analysis using EQS v.6.2 was conducted to test a higher-order three factor model, a correlated three factor model and an uncorrelated three factor model against a single factor model for goodness of fit using the recommended cut-offs for four fit indices: the Satorra-Bentler Chi Square divided by degrees of freedom, the comparative fit index (CFI), the non-normed fit index (NNFI), and the root mean square error of approximation (RMSEA). The fit indices for each model are presented in Table 2. Fit indices indicate good fit of both the correlated and higher order three factor model, with the higher order model (see Figure 1) preferred for its superior fit and mirroring of the factor structure of the original TMSS (Lewis, 2003). Cronbach’s alphas demonstrated that the scales for each of the factors had acceptable internal reliability (specialization 0.68; credibility 0.76; reliability 0.84).

**TABLE 2:** **Fit indices (Robust Statistics) for confirmatory factor analysis models of the TMSS-C (*N* = 397)**.

**Model Cut-off criteria**	**S-B χ^2^/df *p* > 0.05**	**CFI = / > 0.85**	**NNFI =/ > 0.85**	**RMSEA = / < 0.06**
One factor model	*p* < 0.001	0.592	0.524	0.130
Uncorrelated three factor model	*p* < 0.001	0.887	0.868	0.069
Correlated three factor model	*p* < 0.001	0.937	0.924	0.052
Higher order three factor model	0.015*	0.979	0.974	0.030

S-B, Satorra-Bentler; CFI, Comparative Fit Index; NNFI, Non-Normed Fit Index; RMSEA, Root Mean Square Error of Approximation. *The χ^2^ statistic is sensitive to sample size (Kline, 2005). Mean and variance-adjusted Chi Square = 10.479 on 8 df, p = 0.233.

#### Convergent Validity

To assess convergent validity, we calculated correlation coefficients between scores on the TMSS-C subscales and the Expertise Coordination subscales (Faraj and Sproull, 2000). The results are presented in Table 3.

**TABLE 3:** **Convergent validity of the TMSS-C (*n* = 397)**.

	**TMSS-C specialization scale**	**TMSS-C credibility scale**	**TMSS-C coordination scale**
Willingness to assign expertise	0.106*	–0.120*	–0.264**
Willingness to assign expertise to partner	0.042	0.057	–0.121*
Expertise location	0.307**	0.307**	0.311**
Bring expertise to bear	0.175**	0.311**	0.274**

*p < 0.05, **p < 0.01

As expected, the Expertise Location factor was related to the Specialization factor of the TMSS-C (see Table 3), suggesting that both measures captured participants’ ability or willingness to recognize that each member of the relationship had different areas of expertise. This finding further suggests a possible problem with our adaptation of the Knowledge Organization Questionnaire which was intended to capture the same information; perhaps the topics we provided were too specific and not relevant or appropriate for every couple, but participants were able to recognize that each member had different expertise in general, perhaps while considering their own areas of interest. In addition, and as predicted, there was a significant relationship between the Bring Expertise to Bear factor and the Coordination subscale of the TMSS-C, suggesting that both measures captured participants’ use of their expertise. Taken together, these results provide compelling support for the convergent validity of the TMSS-C as an appropriate way to measure the components of transactive memory systems in romantic couples. As Table 3 shows, there were also moderate relationships between each of the two factors of the Expertise Coordination scale, and each of the subscales of the TMSS-C; perhaps these relationships are a result of the high internal consistency of the TMSS-C.

#### Divergent Validity

To assess divergent validity, we calculated correlation coefficients between scores on the TMSS-C subscales and scores on the Self-perceived Communication Competence Scale (McCroskey and McCroskey, 1988), and the Mnemonics Usage factor of the MFQ (Zelinski et al., 1990). As Table 4 illustrates, and as predicted, the TMSS-C subscales were not strongly associated with participants’ self-perceived communication competence, or their reliance on memory aids, thus providing evidence of the divergent validity of the TMSS-C.

**TABLE 4:** **Divergent validity of the TMSS-C (*n* = 397)**.

	**TMSS-C specialization scale**	**TMSS-C credibility scale**	**TMSS-C coordination scale**
Self-perceived communication competence	0.070	0.134*	0.105*
Mnemonic usage	–0.186**	–0.065	–0.009

*p < 0.05, **p < 0.01

## Phase 2—Test–retest Reliability

A smaller sample of 104 participants completed the TMSS-C twice at an interval of 13–15 days to assess the 2-week test–retest reliability. Pearson’s correlations were calculated for each subscale between time 1 and time 2 t (Specialization = 0.69, Credibility = 0.72, and Coordination = 0.78) and indicate the TMSS-C is stable across a 2 week period.

## Summary and Concluding Discussion

Taken together, the results provide support for the initial validation of the TMSS-C as a measure of the components of romantic couples’ transactive memory systems. The higher order three factor model fit the data well and mirrored the factor structure of the original TMSS (Lewis, 2003). We also found reasonable evidence of both convergent validity (against a well-established measure of expertise coordination; Faraj and Sproull, 2000), and of divergent validity, as measured against reliance on memory aids in general (Zelinski et al., 1990) and self-perceived communication competence (McCroskey and McCroskey, 1988). In addition, we found strong evidence of test–retest reliability across a 2-week period. Overall the results provide considerable support for the initial validation of the TMSS-C.

One limitation to this research is that there was no eligibility requirement for both members of a couple to participate, and only 45 intact couples both took part in the research. Of these, the data for only 26 couples was able to be matched. As such, participant’s data was examined at the individual level, and so may not fully capture participants’ transactive memory systems. We acknowledge the inclusion of 26 couples violates the assumption of independence. Further research could recruit both members of existing couples only, and examine data at the dyad level to further establish the validity of the TMSS-C.

The TMSS-C has a number of possible research applications. For instance, the TMSS-C could be used to examine whether a couple’s transactive memory system is associated with other important features of their relationship, such as satisfaction. This type of research could further our understanding of how romantic couples cope with and negotiate everyday tasks and decisions, provide important information about relationship functioning, and might even identify areas for interventions to improve relationship functioning. One difficulty with conducting this type of research is assessing possible risks to the relationship, in addition to possible risks to the individuals, from participating in relationship research. Empirical research into the experiences of romantic couples as research participants is required to guide both researchers’ and ethics committees’ consideration of the costs and benefits of research participation (Newman et al., 2001; Decker et al., 2011). To date, no research has examined the effect of participation in research on transactive memory in couples, and it is not known whether previous findings will generalize to research in this area.

The TMSS-C could also make an important contribution to other research paradigms, especially in the area of memory or task performance. For instance memory conformity research demonstrates that romantic partners are especially likely to incorporate information for each other’s memory reports into their own, reporting seeing things that they never actually saw (French et al., 2008; Hope et al., 2008). One possible explanation of this finding is that romantic couples are already used to relying on each other’s memories through the use of shared transactive memory systems in their relationships, and as a result are also more willing to rely on each other’s memories in recounting past events. The TMSS-C could be incorporated into the memory conformity paradigm to examine whether a stronger or more established transactive memory system is associated with a higher level of memory conformity, whether predictions of memory conformity can be made from one member’s ratings on the TMSS-C, or whether both members need to contribute to elucidate the full picture, and even whether memory conformity might be more or less likely to occur in the members’ specific areas of expertise and non-expertise.

We believe the TMSS-C provides a valuable tool that can quickly and easily capture the components of romantic couples’ transactive memory systems. It has huge potential to help us better understand an intriguing feature of romantic relationships, and may possibly even lead to the development of relationship interventions to improve relationship success.

### Conflict of Interest Statement

The authors declare that the research was conducted in the absence of any commercial or financial relationships that could be construed as a potential conflict of interest.
